# The Diagnosis and Genetic Mechanisms of Prader-Willi Syndrome: Findings From a Moroccan Population Study

**DOI:** 10.7759/cureus.37866

**Published:** 2023-04-20

**Authors:** Mohamed Ahakoud, Hanae Daha Belghiti, Ayoub Nedbour, Abdelhamid Bouramtane, Sana Chaouki, Laila Bouguenouch, Karim Ouldim

**Affiliations:** 1 Medical Genetics and Onco-Genetics Laboratory, Hospital University Hassan II, Fez, MAR; 2 Pediatric Neurology, Hospital University Hassan II, Fez, MAR

**Keywords:** prader-willi syndrome, syndromic mental retardation, fluorescence in situ hybridization (fish), paternal 15q11-q13 deletion, methyl-pcr

## Abstract

Background

Prader-Willi syndrome (PWS) is a complex genetic disorder caused by a deficit in gene expression on the paternal inherited chromosome 15q11.2-q13. It affects various aspects of growth and development, including feeding, cognitive function, and behavior. Early diagnosis and management of PWS can significantly improve outcomes for patients and their families.

Methods

In this study, we analyzed a group of 29 clinically diagnosed patients suspected of PWS. All patients were referred to the medical genetics and onco-genetics service for genetic consultation and molecular analysis. We used DNA methylation analysis and fluorescence in situ hybridization (FISH) to confirm the diagnosis and identify the underlying genetic mechanisms.

Results

Our analysis showed that five out of seven patients (71.43%) with a positive methylation-specific PCR (MSP) had chromosomal deletion by FISH and presented major clinical signs summarized by morbid obesity in 65.21% of cases and neonatal hypotonia in 42.85% of cases. This finding indicates that paternal 15q11-q13 deletion is the most common genetic mechanism involved in PWS.

Conclusion

The results of this study highlight the importance of early diagnosis and molecular analysis in the management of Prader-Willi syndrome. Our findings contribute to a better understanding of the genotype-phenotype correlation in the Moroccan population and provide families with a rigorous molecular diagnosis, relevant genetic counseling, and multidisciplinary support. Further research is needed to explore the underlying mechanisms of PWS and develop effective interventions to improve outcomes for affected individuals.

## Introduction

Prader-Willi syndrome (PWS) is a complex and rare genetic disorder that has garnered significant attention from the scientific community due to its diverse clinical manifestations and potential impact on the affected individual's quality of life. The disorder is caused by abnormalities in the q11-13 region on the paternal chromosome 15 and is known to affect approximately one in 15,000 to one in 25,000 individuals worldwide, with an average mortality rate of 3% [1-3]. PWS is characterized by a range of clinical features, including hypotonia, feeding difficulties, developmental delays, short stature, abnormal neonatal behavior, obesity, and poor hypothalamic and gonadal development [4]. Due to the multifaceted nature of PWS, early diagnosis is essential for implementing comprehensive treatment strategies that aim to improve growth and minimize developmental disorders, ultimately enhancing the patient's quality of life and long-term outcomes.

The etiology of PWS can be attributed to the loss of function of imprinted genes located in the 15q11-13 region on the paternal chromosome, leading to genomic imprinting and uniparental disomy. In most cases, the disorder arises from a deletion in the paternal chromosome, while maternal uniparental disomy accounts for a smaller percentage of cases. Furthermore, some cases of PWS are caused by imprinting defects or chromosomal translocations [5]. Understanding the underlying genetic mechanisms of the syndrome is vital for accurate diagnosis and the development of targeted therapeutic interventions.

Molecular techniques, such as DNA methylation analysis and fluorescence in situ hybridization (FISH), have become instrumental in the diagnosis and identification of the genetic mechanisms involved in PWS. DNA methylation analysis can determine the presence of an abnormal methylation pattern specific to PWS, while FISH allows for the visualization of chromosomal abnormalities, such as deletions or translocations, which may contribute to the development of the syndrome. Employing these methods in a clinical setting facilitates prompt and accurate diagnosis, enabling healthcare providers to develop and implement tailored treatment plans for affected individuals.

The impact of PWS on patients extends beyond the physical manifestations of the disorder, often affecting their cognitive and behavioral development as well. As such, it is essential to adopt a multidisciplinary approach to the management of PWS that encompasses medical, nutritional, and behavioral interventions [6]. Early intervention can optimize growth, prevent obesity, and address developmental and behavioral challenges, ultimately improving the patient's overall prognosis. Comprehensive treatment plans may include growth hormone therapy, nutritional counseling, physical therapy, and psychological support, all of which aim to enhance the patient's quality of life and mitigate the impact of PWS on their daily functioning [7].

Growth hormone therapy has emerged as a promising intervention for children with PWS, as it can significantly improve growth velocity and weight gain. Studies have demonstrated that long-term growth hormone therapy can change the natural history of body composition and motor function in children with PWS [7]. Moreover, the Growth Hormone Research Society has established consensus guidelines for recombinant human growth hormone therapy in PWS, ensuring that this treatment is administered safely and effectively [8]. By incorporating growth hormone therapy into comprehensive treatment plans, healthcare providers can help patients with PWS achieve better growth outcomes, which may improve their overall quality of life.

In addition to growth hormone therapy, nutritional management is a critical component of PWS treatment [6]. Individuals with PWS often exhibit hyperphagia, or excessive hunger, which can lead to obesity and associated comorbidities if left unaddressed [9]. Therefore, it is crucial to establish early dietary interventions and ongoing nutritional counseling to prevent excessive weight gain and promote optimal health in individuals with PWS. This may include the implementation of calorie-restricted diets, portion control, and regular monitoring of weight and growth [10].

Moreover, addressing the behavioral and emotional challenges associated with PWS is an essential aspect of comprehensive treatment. Patients may experience a range of psychiatric symptoms, including anxiety, obsessive-compulsive tendencies, and mood disturbances. These challenges can significantly impact an individual's social functioning and overall well-being. Early intervention with targeted behavioral therapies, counseling, and psychopharmacological management can help mitigate these symptoms and improve the patient's quality of life [11].

The current study aims to evaluate the clinical manifestations of PWS in 29 patients suspected of having the syndrome. These patients were referred to the medical genetics and onco-genetics service for genetic consultation and molecular analysis. The use of DNA methylation analysis and FISH in this study will not only confirm the diagnosis of PWS but also help identify the underlying genetic mechanisms involved. This study will add to the understanding of PWS by exploring its diverse clinical symptoms and genetic causes. This information will be valuable for future research in the field.

## Materials and methods

Study design

The study employed a retrospective approach, conducted in the medical genetics and pediatrics department of Centre Hospitalier Universitaire (CHU) Hassan II in Fez over a five-year period (January 2017 to December 2021), which included seven cases of patients diagnosed with PWS.

Study population

The study population comprised patients who were confirmed to have PWS through molecular biology. Patients were retrospectively included if they fulfilled the following inclusion criteria: received care between 2017 and 2021, presented with a clinically suspected diagnosis, and received genetic confirmation. Exclusion criteria encompassed patients with PWS-like symptoms, monogenic obesity, and suspected PWS without genetic confirmation.

Patient recruitment sources

To compile the list of study participants, we utilized the archive of the medical genetics and pediatrics department of CHU Hassan II Fez and the Hospital Information System (HOSIX).

Genetic diagnosis

All patients underwent genetic confirmation using a methyl PCR method, followed by FISH to identify the molecular mechanism involved.

DNA extraction

Genomic DNA extraction from blood lymphocytes was performed using the INVITROGEN PureLink Genomic DNA extraction kit from Thermo Fisher Scientific (Waltham, MA, USA), with steps including the addition of proteinase K and RNase, sample lysis, column washing, and purified DNA elution.

Bisulfite treatment

The EPI JET Bisulfite Conversion Kit K1461 from Thermo Fisher Scientific was employed to obtain bisulfite-converted DNA. The procedure entailed modifying genomic DNA with sodium bisulfite and implementing two sets of primers for PCR (Table 1).

**Table 1:** Primers used to amplify target regions of the SNRPN gene in its methylated and unmethylated state SNRPN, small nuclear ribonucleoprotein polypeptide N; SNRPNM, mutated small nuclear ribonucleoprotein polypeptide N

Gene	Primer sequence	Primer type	Temperature (°C)
SNRPN	TAAATAAGTACGTTTGCGCGGTC	F	60
	AACCTTACCCGCTCCATCGCG	R	
SNRPNM	GTAGGTTGGTGTGTATGTTTAGCT	F	60
	ACATCAAACATCTCCAACAACCA	R	

Reagents and DNA storage

Reagents were supplied as a dry mix and required dissolution before use. Bisulfite-converted DNA was stored at -80°C, -20°C, or 4°C for up to six months, three months, and 1-2 weeks, respectively.

PCR product visualization

DNA fragments were separated using electrophoresis. Ethidium bromide, which fluoresces red when exposed to UV rays, was employed to visualize the separated fragments as bands. The methylated DNA from the positive control should exhibit an amplicon of appropriate size for the M primer series, while the non-methylated DNA from the positive control should display an amplicon of appropriate size for the U primer series.

FISH

FISH leverages the properties of DNA to denature and renature under controlled conditions, enabling the detection of specific chromosomal regions. This technique necessitates a labeled DNA probe and the patient's chromosomes as targets. Prior to hybridization, a cell culture step is essential for obtaining metaphase cells. The complex preparation process for hybridization involves steps such as centrifugation, fixation, spreading, dehydration, and probe mix preparation.

The Vysis Prader-Willi/Angelman Region Probe-LSI GABRB3 Spectrum Orange/CEP 15 (D15Z1) Spectrum Green was used as the probe, which hybridizes to chromosome 15q11-13 (Spectrum Orange GABRB3) and the satellite III region (15p11.2 band region, locus D15Z1) of human chromosome 15. Probe hybridization facilitates the detection of copy number anomalies for the tested region. The hybridized probe emits moderate to strong fluorescence in interphase nuclei and on metaphase chromosomes.

## Results

Case 1

A 10-year-old male with obesity presented with neonatal hypotonia, delayed psychomotor development, and a family history of toxic goiter. Weighing 60 kg and 129 cm tall, he had a body mass index (BMI) of 36.1 kg/m^2^. Clinical examination showed mild facial dysmorphism, cryptorchidism, and an absent right testicle. Biological assessments were mostly normal, but elevated gamma-glutamyl transferase (GGT) (123 UI/L) and alkaline phosphatase (ALP) (144 UI/L) indicated potential liver issues. A hormonal assessment revealed low estradiol (<10 pg/mL). Ultrasound confirmed the left pelvic testicle and the absent right testicle. Genetic testing at age 5 indicated uniparental contribution and Prader-Willi syndrome diagnosis. FISH detected a 15q11-13 locus deletion in 100 analyzed nuclei. The patient underwent right testicular ectopia surgery at age 4.

Case 2

A nine-year-old female with potential obesity and a family history of diabetes experienced neonatal hypotonia, seizures, and drug rash. She weighed 26 kg, was 84 cm tall, and had a BMI of 36.8 kg/m^2^. Clinical examination showed facial dysmorphism and brachydactyly. Biochemical analysis and hormonal tests were mostly normal, with elevated adrenocorticotropic hormone (ACTH) of 32.2 pg/mL. Genetic testing at age 7 indicated uniparental contribution and Prader-Willi syndrome diagnosis. FISH revealed no 15q11-13 locus deletion in 100 analyzed nuclei. The patient exhibited aggravated neurological symptoms and persistent seizures despite antiepileptic treatment.

Case 3

An eight-year-old female with neonatal hypotonia and delayed psychomotor development presented with a BMI of 35.5 kg/m^2^, bilateral strabismus, and lumbar issues. Biochemical analysis showed normal values. Genetic testing at age 5 revealed uniparental contribution and a Prader-Willi syndrome diagnosis. FISH detected a 15q11-13 locus deletion in 100 analyzed nuclei. The patient exhibited good psychomotor development progression with reinforced follow-up.

Case 4

A five-year-old male with delayed psychomotor development and learning difficulties had a BMI of 22 kg/m^2^, facial dysmorphism, and a micropenis. Biochemical analysis was mostly normal, with low high-density lipoprotein (HDL) cholesterol. The radiological assessment showed no visualization of the left testicle and multiple lymph nodes. Genetic testing at age 5 revealed uniparental contribution and Prader-Willi syndrome diagnosis. FISH detected a 15q11-13 locus deletion in 100 analyzed nuclei. The patient had surgery for the left ectopic testicle at age 3.

Case 5

A 15-year-old male with neonatal hypotonia and obesity had a BMI of 34.7 kg/m^2^, a thin upper lip, and a micropenis. The biological assessment showed mostly normal values. Genetic testing at age 11 revealed uniparental contribution and Prader-Willi syndrome diagnosis. FISH detected deletion in the 15q11-13 locus in 100 analyzed nuclei. The patient experienced sleep apnea and was under pneumologist follow-up.

Case 6

A six-year-old male with delayed psychomotor development, learning difficulties, and language impairment presented with a BMI of 34.9 kg/m^2^ and micromelia. Biochemical analysis revealed anemia and normal liver function tests. Genetic testing at age 5 revealed uniparental contribution and Prader-Willi syndrome diagnosis. FISH detected a 15q11-13 locus deletion in 100 analyzed nuclei. The patient received pediatric psychiatric care for learning and language difficulties.

Case 7

A 16-year-old female with delayed menarche, neonatal hypotonia, delayed speech, and psychomotor developmental delay had a BMI of 35.7 kg/m^2^. Genetic testing at age 15 confirmed Prader-Willi syndrome, without deletion in the 15q11-13 locus found by FISH. The patient experienced respiratory issues, such as sleep apnea.

The clinical characteristics of the above patients are shown in Table 2.

**Table 2:** Main clinical characteristics of PWS-positive patients PWS, Prader-Willi syndrome

Observation number	Age (at genetic diagnosis of PWS)	Gender	Hypotonia	Eating disorders	Hyperphagia	Obesity	Intellectual disability	Facial dysmorphism	Hypogonadism	Hypopigmentation	Consensus: diagnostic criteria
1	5	Male	+	+	+	+	+	+	+	-	7/8
2	7	Female	+	+	+	+	-	+	-	-	5/8
3	5	Female	+	-	-	+	+	+	-	-	4/8
4	5	Male	+	-	-	-	+	+	+	-	4/8
5	11	Male	+	+	+	+	-	+	+	-	6/8
6	5	Male	+	+	+	+	+	+	-	-	6/8
7	15	Female	+	+	+	+	+	+	+	-	7/8

## Discussion

The interest in PWS, also known as "Prader-Labhart-Willi syndrome," is relatively recent. Doctors Prader, Labhart, Willi, and Franconi first described it in 1956. It was not until 1981 that Dr. Ledbetter was able to isolate a deletion on chromosome 15 in his patients, and it was not until 1989 that molecular analyses revealed that the deletion occurred on the paternal chromosome. In that same year, maternal disomy of this same chromosome was recognized as a second possible genetic form of this syndrome [12]. The prevalence of PWS is estimated to be approximately one in 15,000-20,000 births [13]. This prevalence appears to be consistent regardless of sex, geographic origin, or socioeconomic status [14]. The risks of recurrence for future pregnancies are very rare and depend on the specific chromosomal anomaly involved. When the disease is caused by a de novo microdeletion or non-parental uniparental disomy, the risk of recurrence is similar to that of the general population (less than 1%). However, in cases associated with familial genetic anomalies (translocation, mutation of the genetic imprinting center, or deletion on paternal chromosomes), the risk of recurrence is higher, ranging from 25% to 50% [12].

Genetic mechanisms

**Figure 1:**
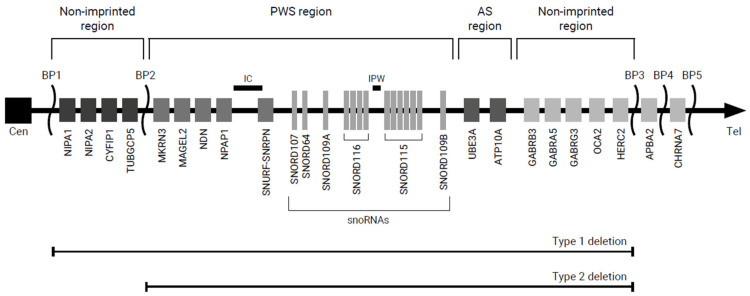
Genetic map of the critical region of Prader-Willi syndrome on chromosome 15 AS, Angelman syndrome; BP, breakpoint; Cen, centromere; IC, imprinting center; PWS, Prader-Willi syndrome; Tel, telomere Used with permission from [15]

The cause of PWS is a genetic rearrangement, usually accidental, that leads to the absence or loss of function of genes located in the q11-q13 region of the paternal copy of chromosome 15. In PWS, maternal alleles are silent, and it is the lack of expression of paternal alleles that allows the disease to be expressed. This corresponds to the phenomenon of parental genomic imprinting, where the alleles of genes located in the 15q11-q13 region do not have the same functions on maternal and paternal chromosomes, but both are required for normal development (Figure 1).

Three types of anomalies lead to the non-expression of these genes. The first type is a chromosomal microdeletion (65% of cases) or absence of the q11-q13 region on the paternal chromosome 15. Two types of deletions have recently been identified, type I and type II, with type I being the longer of the two. The second type of anomaly is maternal uniparental disomy (30% of cases) or absence of the paternal chromosome 15 (at least in the q11-q13 region) but with two copies of the maternal chromosome. The third type of anomaly is a mutation in the genetic imprinting center on the paternal chromosome 15, which renders the genes inactive (1%-5% of cases) [3].

Diagnosis

Clinical

The diagnosis of PWS should be considered in the neonatal period in the presence of any severe and unexplained hypotonia or later on in the case of morbid obesity in a hyperphagic child. Early diagnosis is indeed an important factor influencing the outcome of these children. The aforementioned author has conducted significant awareness work, especially among neonatologists, and as a result, the vast majority of children in France are currently diagnosed in the neonatal period, with a median age of diagnosis of two months [16]. In our study, the average age of patients at the time of diagnosis was six years and eight months. Furthermore, it was observed that males are more susceptible to Prader-Willi syndrome, accounting for 51.73% of cases, while the female sex represents only 48.27% of cases. Nonetheless, the prevalence of this syndrome in the general population is 1:1.

New diagnostic recommendations have been released for PWS, taking into account clinical variability and age-related changes. To diagnose PWS in neonates, severe and unexplained hypotonia should be investigated, and dysmorphic signs are highly suggestive of the condition (almond-shaped eyes, thin upper lip, small hands and feet, etc.). For children, the diagnosis should be considered if they are obese and have learning difficulties, stunted growth, or dysmorphic features. In adolescents and adults, PWS should be considered if there are behavioral and psychiatric disorders accompanied by obesity and pubertal developmental disorders [17].

Hypotonia: Hypotonia is observed in approximately 97% of newborns with PWS. In utero, the first signs are reduced fetal movement, also known as decreased fetal activity [18], and hydramnios in the third trimester is present in 31.6% of cases [19].

Hypotonia in these newborns may lead to hypoventilation and acute respiratory infections, both of which may contribute to sudden infant death. Additionally, hypotonia in the neonatal period is associated with feeding difficulties, including issues with sucking and swallowing, often requiring tube-feeding techniques in the first few months of life [19].

Hypotonia gradually decreases after eight months of age. However, postural problems usually persist due to muscle weakness and joint instability. Tonic problems also have adverse secondary effects on motor development, exploratory behaviors, and speech, such as hypotonia in the facial and oral muscles [20].

Stature: Small stature is a common trait in 90%-95% of cases, and 20%-30% of cases experience intrauterine growth retardation in childhood, with birth height usually normal or slightly below average. Growth retardation becomes noticeable around the age of one year after a first slowdown in growth during the first few months of life. Small stature is further exacerbated by the absence of the pubertal growth spurt, and a new break in the growth curve occurs during adolescence. The delay in height is at least partially due to a deficiency in growth hormone, and treatment with exogenous growth hormone can accelerate children's growth and improve adult height [21,22].

Weight: Obesity has long been considered inevitable and incurable in this condition, and in the absence of early intervention, it is observed in almost all cases. While birth weight is usually reduced (-0.87 standard deviation (SD) for girls and -1.17 SD for boys), weight gain becomes very excessive between the ages of one and six. This can be falsely reassuring for those around the child, given that the child had up until then had feeding difficulties [9].

In the context of SPW, obesity is very different from common obesity. On the one hand, it is associated with stature delay or a slowdown in growth rate. On the other hand, it is established as early as the second year of life and progresses very rapidly. Additionally, lean mass is low, and the distribution of fat is unique, with a disposition at the level of the face, abdomen, hips, buttocks, and thighs [23].

Obesity in this context is both the result of excess calorie intake and a decrease in expenditure. The latter is reduced due to lower basic energy expenditures (basal metabolism is reduced due to low muscle mass) and especially due to insufficient expenditure related to physical activity (hypotonia and hypoactivity). This results in significantly lower caloric requirements (10%-20%) than those of the general population [24].

The consequences of this obesity are multiple. Medically, it can lead to arterial hypertension, as well as cardiovascular, hepato-gastrointestinal, respiratory (sleep apnea), osteoarticular, static disorders, worsening scoliosis, metabolic, and endocrine complications (increased frequency of diabetes) [19]. Thus, obesity reduces life expectancy and is the main cause of mortality in individuals with PWS. Hyperphagia can also directly lead to death, through gastric dilation with necrosis and perforation. Beyond these medical impacts, obesity also carries significant psychosocial and emotional consequences (self-esteem, self-image, gaze of others, and difficulties in social relationships) that should not be neglected [20].

Since the discovery of PWS, attempts have been made to establish a correlation between body mass index (BMI) and intelligence quotient (IQ). However, no significant correlation has been found, as evidenced by research studies [25]. Our own study has also shown no correlation between the presence of morbid obesity.

In recent years, there has been extensive research into the genetic basis of PWS. However, clinical diagnosis of this disease remains complex, as certain characteristics may change with age and may also be present in other syndromes. In newborns, the clinical characteristics of PWS include lack of crying, feeding difficulties, and neonatal hypotonia [26], which we observed in our study in 31.04% of cases.

Pubertal development and hypogonadism: The term "congenital hypogonadism" refers to a complete or partial pubertal insufficiency due to insufficient secretion of the pituitary gonadotropins luteinizing hormone (LH) and follicle-stimulating hormone (FSH) and gonadal steroid hormones. Hypogonadism is a common clinical feature in PWS. Hypoplasia of the genitalia in females and micropenis with hypoplastic scrotum in males are evident at birth. Unilateral or bilateral cryptorchidism is present in 80%-90% of males [27].

Other conditions: Individuals with PWS may also exhibit a high pain threshold, facial dysmorphia, and bone and orthopedic problems, as well as respiratory, ophthalmological, dermatological, and thermoregulatory abnormalities [27].

Developmental delay and learning difficulties: In the context of PWS, all developmental spheres are affected by a delay in motor and language development, cognitive processes, and adaptive behaviors [27].

Psychomotor retardation: Psychomotor developmental delay is observed in 90%-100% of cases and affects both gross and fine motor skills. It is thought to be more related to anomalies of the central nervous system than to muscular disorders [27]. This delay is manifested early and is exacerbated by severe hypotonia in the first few months of life.

Language, expression, and communication difficulties: Language development is often difficult and delayed in individuals with PWS. Of children with this syndrome, 93% have a language level lower than what would be expected given their IQ and age. As an indication, the first words appear around 18-24 months, and the first sentences appear around three years old. Syntax can also be an obstacle to verbal expression for these individuals. The sentences are imperfectly constructed, incomplete, and lacking in complex structures (use of tenses, passive forms, etc.). Syntax errors would be comparable to difficulties experienced by young children (difficulties that are normal but persist when they should not) [28].

Such difficulties in articulation and sound production likely result from a combination of various factors encountered in the context of PWS, such as cerebral dysfunctions, the particular anatomy of the larynx and mouth, cognitive delay, and hypotonia of the buccal, facial, and palatal muscles, as well as saliva viscosity and even obesity [28].

Genetic

**Figure 2:**
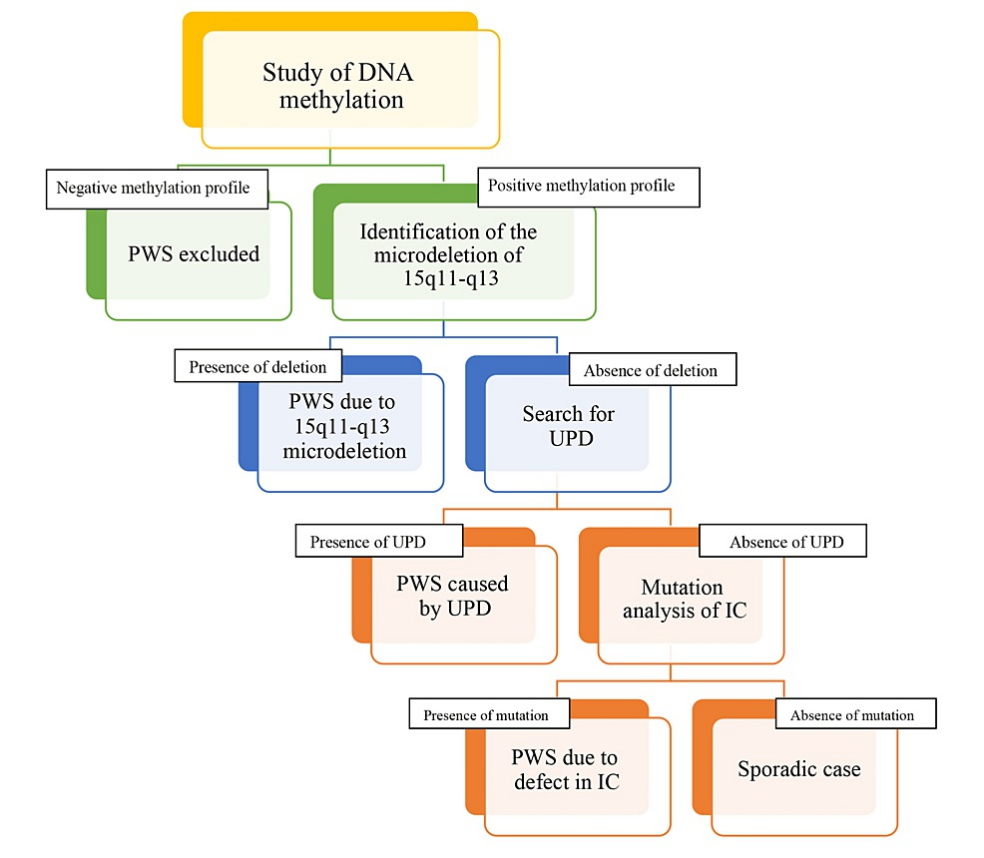
Algorithm for the diagnosis of Prader-Willi syndrome PWS, Prader-Willi syndrome; DNA, deoxyribonucleic acid; UPD, uniparental disomy; IC, imprinting center

For PWS, diagnosis is based on clinical signs, but confirmation is nowadays based on genetic analysis. Genetic confirmation of the syndrome relies mainly on the detection of an abnormal DNA methylation profile by molecular biology or on the detection of deletion by fluorescence in situ hybridization (FISH) (Figure 2).

Methyl PCR: Analysis of DNA methylation is the most effective way to confirm the diagnosis of PWS in cases of clinical suspicion. DNA methylation is a powerful tool to assess paternal-only, maternal-only, or biparental (normal) contribution.

Normal individuals have both a methylated and an unmethylated allele, whereas individuals with PWS only possess the methylated maternal allele. This makes methylation-specific PCR (MSP) the most effective analysis for diagnosing PWS. The most widely used DNA methylation analysis targets only the CpG islands of the 5′ SNRPN locus and can correctly diagnose PWS in more than 99% of cases; however, it cannot distinguish between deletion, uniparental disomy, or imprinting center defect.

Of the 29 cases collected, seven (24.13%) patients had a positive methylation and 22 (75.86%) patients had a negative methylation.

**Figure 3:**
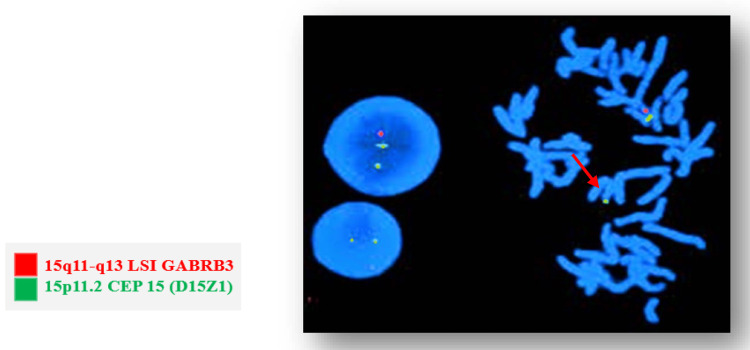
Microscopic observation of microdeletion of the 15q11-q13 region

FISH: According to the PWS diagnostic algorithm, a diagnosis is made when a positive methylation profile supports the suspected syndrome and confirms the presence of two maternal alleles. If a microdeletion is not detected in such a case, the clinical diagnosis of the syndrome is still considered, and analysis is directed toward detecting uniparental disomy (Figure 3).

The absence of microdeletion by FISH does not exclude the diagnosis of PWS when the methylation profile is abnormal. In cases of strong clinical suspicion, molecular genetic testing is necessary to confirm the diagnosis.

Screening for PWS in cases where microdeletion was absent can be divided into two categories: analysis of microsatellites to detect uniparental disomy and other specific analyses using various molecular techniques to identify mutations leading to imprinting defects.

Treatment

As of today, there is no cure for PWS. The only available treatment involves minimizing certain consequences to improve the quality of life for patients and their families. The somatic, psychological, and social repercussions of this syndrome require integrated responses based on early and multidisciplinary care. Such care is crucial in supporting and preventing many complications. The management of SPW must, therefore, be multidisciplinary and adapted to the evolving nature of the syndrome. The main stages of care are as follows: from birth to three years old: monitoring of growth and weight, dietary management, speech therapy (to reduce tube feeding duration and facilitate language development), physiotherapy, growth hormone therapy (initiated at around one year of age), parental guidance, and family support; from 10 to 12 years old: prevention of obesity, monitoring of body composition, promotion of physical activity (to improve hypotonia and prevent obesity), speech and language therapy, identification of factors favoring sleep apnea and polysomnography, growth hormone therapy, monitoring of learning and schooling, and psychological support for the child and family; and during puberty: weight control, surgical management of scoliosis if necessary, identification of sleep apnea or daytime sleepiness, reassessment of growth hormone secretion, and induction of puberty with hormones.

The goals of care are to evaluate patients' evolution and detect any potential complications (somatic and psychological) to provide appropriate management and improve the quality of life of patients and their families. These evaluations throughout the patient's life allow for the readjustment of various medical, pharmacological, and paramedical interventions if necessary.

While our study provides valuable insights into PWS and its clinical characteristics, it is important to consider the limitations of the study. First, the sample size of our study is relatively small, which may limit the generalizability of the results to the broader PWS population. Larger-scale studies would be beneficial to better understand the prevalence and clinical manifestations of PWS [29]. Second, the retrospective nature of the study may introduce bias, as the data collected may be subject to inconsistencies or inaccuracies in medical records. A prospective study design, in which patients are followed over time, would help to minimize these biases [30]. Third, our study may not fully capture the range of clinical manifestations of PWS, as certain characteristics may change with age and may also be present in other syndromes. More comprehensive studies that account for these factors would provide a more accurate understanding of the clinical characteristics of PWS. Finally, our study focuses on the genetic basis of PWS and its clinical diagnosis but does not explore the potential environmental or epigenetic factors that may influence the development and progression of the syndrome. Future research should consider these factors to gain a more holistic understanding of PWS and its underlying mechanisms.

## Conclusions

In conclusion, this study emphasizes the significance of early diagnosis and molecular analysis in the management of Prader-Willi syndrome, a complex genetic disorder affecting various aspects of growth and development. The use of DNA methylation analysis and fluorescence in situ hybridization helped confirm the diagnosis and identify the underlying genetic mechanisms. Our findings indicate that paternal 15q11-q13 deletion is the most common genetic mechanism involved in PWS in the Moroccan population. This study contributes to a better understanding of the genotype-phenotype correlation and provides families with a rigorous molecular diagnosis, relevant genetic counseling, and multidisciplinary support. Further research is needed to explore the underlying mechanisms of PWS and develop effective interventions to improve outcomes for affected individuals.

## References

[1] Cassidy SB, Driscoll DJ (2009). Prader-Willi syndrome. Eur J Hum Genet.

[2] Whittington J, Holland A (2010). Neurobehavioral phenotype in Prader-Willi syndrome. Am J Med Genet C Semin Med Genet.

[3] Irizarry KA, Miller M, Freemark M, Haqq AM (2016). Prader Willi syndrome: genetics, metabolomics, hormonal function, and new approaches to therapy. Adv Pediatr.

[4] Goldstone AP, Holland AJ, Hauffa BP, Hokken-Koelega AC, Tauber M (2008). Recommendations for the diagnosis and management of Prader-Willi syndrome. J Clin Endocrinol Metab.

[5] Horsthemke B, Buiting K (2006). Imprinting defects on human chromosome 15. Cytogenet Genome Res.

[6] Miller JL, Lynn CH, Driscoll DC (2011). Nutritional phases in Prader-Willi syndrome. Am J Med Genet A.

[7] Carrel AL, Myers SE, Whitman BY, Eickhoff J, Allen DB (2010). Long-term growth hormone therapy changes the natural history of body composition and motor function in children with prader-willi syndrome. J Clin Endocrinol Metab.

[8] Deal CL, Tony M, Höybye C, Allen DB, Tauber M, Christiansen JS (2013). Growth Hormone Research Society workshop summary: consensus guidelines for recombinant human growth hormone therapy in Prader-Willi syndrome. J Clin Endocrinol Metab.

[9] Holland A, Whittington J, Hinton E (2003). The paradox of Prader-Willi syndrome: a genetic model of starvation. Lancet.

[10] Heksch R, Kamboj M, Anglin K, Obrynba K (2017). Review of Prader-Willi syndrome: the endocrine approach. Transl Pediatr.

[11] Burman P, Ritzén EM, Lindgren AC (2001). Endocrine dysfunction in Prader-Willi syndrome: a review with special reference to GH. Endocr Rev.

[12] Goldstone AP (2004). Prader-Willi syndrome: advances in genetics, pathophysiology and treatment. Trends Endocrinol Metab.

[13] Butler JV, Whittington JE, Holland AJ, Boer H, Clarke D, Webb T (2002). Prevalence of, and risk factors for, physical ill-health in people with Prader-Willi syndrome: a population-based study. Dev Med Child Neurol.

[14] Diene G, Mimoun E, Feigerlova E, Caula S, Molinas C, Grandjean H, Tauber M (2010). Endocrine disorders in children with Prader-Willi syndrome--data from 142 children of the French database. Horm Res Paediatr.

[15] Holland AJ, Aman LC, Whittington JE (2019). Defining mental and behavioural disorders in genetically determined neurodevelopmental syndromes with particular reference to Prader-Willi syndrome. Genes (Basel).

[16] Méndez-Rosado LA, García D, Molina-Gamboa O, García A, de León N, Lantigua-Cruz A, Liehr T (2020). Algorithm for the diagnosis of patients with neurodevelopmental disorders and suspicion of a genetic syndrome. Arch Argent Pediatr.

[17] Yang L, Zhou Q, Ma B, Mao S, Dai Y, Zhu M, Zou C (2020). Perinatal features of Prader-Willi syndrome: a Chinese cohort of 134 patients. Orphanet J Rare Dis.

[18] Haig D, Wharton R (2003). Prader-Willi syndrome and the evolution of human childhood. Am J Hum Biol.

[19] Schrander-Stumpel CT, Curfs LM, Sastrowijoto P, Cassidy SB, Schrander JJ, Fryns JP (2004). Prader-Willi syndrome: causes of death in an international series of 27 cases. Am J Med Genet A.

[20] Abdilla Y, Andria Barbara M, Calleja-Agius J (2017). Prader-Willi syndrome: background and management. Neonatal Netw.

[21] Glattard M (2012). Psychological, cognitive and behavioral aspects of children with Prader-Willi syndrome: cross-sectional study and longitudinal study (Book in French). https://theses.hal.science/tel-00718614/.

[22] Tan Q, Orsso CE, Deehan EC (2020). Current and emerging therapies for managing hyperphagia and obesity in Prader-Willi syndrome: a narrative review. Obes Rev.

[23] Damiano J, Ficko C, Garrabé E (2003). [Extreme obesity in Prader-Willi Syndrome (PWS)] (Article in French). Rev Med Interne.

[24] Schalock RL, Luckasson R (2004). American Association on Mental Retardation's Definition, Classification, and System of Supports and its relation to international trends and issues in the field of intellectual disabilities. J Policy Pract Intellect Disabil.

[25] Bass JL, Corwin M, Gozal D (2004). The effect of chronic or intermittent hypoxia on cognition in childhood: a review of the evidence. Pediatrics.

[26] Denizot S, Boscher C, Le Vaillant C, Rozé JC, Gras Le Guen C (2004). Distal arthrogryposis and neonatal hypotonia: an unusual presentation of Prader-Willi syndrome (PWS). J Perinatol.

[27] Angulo MA, Butler MG, Cataletto ME (2015). Prader-Willi syndrome: a review of clinical, genetic, and endocrine findings. J Endocrinol Invest.

[28] Boer H, Holland A, Whittington J, Butler J, Webb T, Clarke D (2002). Psychotic illness in people with Prader Willi syndrome due to chromosome 15 maternal uniparental disomy. Lancet.

[29] Cassidy SB, Schwartz S, Miller JL, Driscoll DJ (2012). Prader-Willi syndrome. Genet Med.

[30] Mann CJ (2003). Observational research methods. Research design II: cohort, cross sectional, and case-control studies. Emerg Med J.

